# INTEGRATIVE MULTI-OMICS ANALYSIS REVEALS THE CRITICAL ROLE OF PBXIP1 GENE IN AGING-RELATED ALZHEIMER'S DISEASE

**DOI:** 10.1093/geroni/igac059.2209

**Published:** 2022-12-20

**Authors:** Zuyun Liu, Jingyun Zhang, Xiaoyi Sun

**Affiliations:** Zhejiang University School of Medicine, Hangzhou, Zhejiang, China (People's Republic); Zhejiang University School of Medicine, Hangzhou, Zhejiang, China (People's Republic); Zhejiang University School of Medicine, Hangzhou, Zhejiang, China (People's Republic)

## Abstract

Alzheimer’s disease (AD) is a neurodegenerative disorder, and its strongest risk factor is aging. A few studies have explored the relationship between aging and AD, while the underlying mechanism remains unclear. We assembled data cross multi-omics (i.e., epigenetics, transcriptomics, and proteomics, based on frozen tissues from the dorsolateral prefrontal cortex) and neuropathological and clinical traits from the Religious Orders Study and Rush Memory and Aging Project (ROSMAP). Aging was assessed using six epigenetic clocks (including Horvath clock, Hannum clock, Levine clock, Horvathskin clock, Lin clock, and Cortical clock) that capture mortality risk in literature. After accounting for age, we first identified a gene module (including 263 genes) that was related to most epigenetic clocks (e.g., P=3.61×10−5 for Levine clock) and three neuropathological traits of AD (i.e., β-amyloid, Tau tangles, and tangle density). Interestingly, among 20 key genes with top intramodular connectivity of the module, PBXIP1 was the only one that was significantly associated with all three neuropathological traits of AD at the protein level after Bonferroni correction. Furthermore, PBXIP1 was associated with clinical diagnosis of AD in both ROSMAP and two independent datasets. The results suggest the critical role of PBXIP1 in aging-related AD and support the potential and feasibility of using multi-omics data to investigate mechanisms of complex diseases. However, more validations in different populations and experiments in vitro and in vivo are required in the future.

